# Anti-Inflammatory and Antinociceptive Activity of Pollen Extract Collected by Stingless Bee *Melipona fasciculata*

**DOI:** 10.3390/ijms20184512

**Published:** 2019-09-12

**Authors:** Alberto Jorge Oliveira Lopes, Cleydlenne Costa Vasconcelos, Francisco Assis Nascimento Pereira, Rosa Helena Moraes Silva, Pedro Felipe dos Santos Queiroz, Caio Viana Fernandes, João Batista Santos Garcia, Ricardo Martins Ramos, Cláudia Quintino da Rocha, Silvia Tereza de Jesus Rodrigues Moreira Lima, Maria do Socorro de Sousa Cartágenes, Maria Nilce de Sousa Ribeiro

**Affiliations:** 1 Experimental Study of Pain Laboratory, Department of Physiological Sciences, Federal University of Maranhão, São Luís 65080-805, Brazil; cleydlenne@yahoo.com.br (C.C.V.); lenna1911@hotmail.com (R.H.M.S.); pedrofelipe.queiroz@gmail.com (P.F.d.S.Q.); caiovianafernandes@outlook.com (C.V.F.); jbgarcia@uol.com.br (J.B.S.G.); silviaterezam@gmail.com (S.T.d.J.R.M.L.); 2 Laboratory of Pharmacognosy, Department of Pharmacy, Federal University of Maranhão, São Luís 65080-805, Brazil; franciscopho2015@gmail.com; 3 Research Laboratory Information Systems, Department of Information, Environment, Health and Food Production, Federal Institute of Piauí, Teresina 64000-040, Brazil; ricardo@ifpi.edu.br; 4 Chemistry Department, Federal University of Maranhão, São Luís 65080-805, Brazil; claudiarocha3@yahoo.com.br

**Keywords:** pollen, extract, pain, anti-inflammatory, natural products, molecular docking

## Abstract

The stingless bee, *Melipona fasciculata* Smith (Apidae, Meliponini), is a native species from Brazil. Their products have high biotechnological potential, however there are no studies about the biological activities of pollen collected by *M. fasciculata*. In this context, the present study investigated the chemical composition, anti-oxidant, anti-inflammatory, and analgesic activities of hydroethanolic pollen extracts collected by *M. fasciculata* in three cities in Maranhão State, Brazil. We verified the antioxidant activity of the extracts and inhibitory activity against the cyclooxygenase enzyme using in vitro assays and in allowed to select the extract with higher efficiency to be used on in vivo assays. In these trials, the selected extract showed high anti-inflammatory activity as well as nociceptive effects at central and peripheral level, suggesting that this extract acts on inhibition of histamine release and decreased synthesis of prostaglandins and the in-silico study suggested that polyphenols and acids fatty acids in the extract may be associated with these activities. The results of the present study report the high biological potential of pollen extract and we conclude that the pollen collected by *M. fasciculata* can be considered as the object of research for new pharmacological alternatives.

## 1. Introduction

Natural products are sources of discovery and development of drugs to treat different diseases for centuries. The search for increasingly powerful and less toxic molecules is constant, where secondary metabolites, especially from medicinal plants, are a promising source for the selection of compounds with pharmacological interest [1,2,3,4]. In this context, natural bee products such as honey, propolis, geopropolis, wax, royal jelly, and pollen have been extensively employed since ancient times due to their wide pharmacological and nutritional activity [5]; thus demonstrating high biological potential which justifies its use for the development of new drugs.

Stingless bee products have long been used in herbal medicine and diet for their positive health consequences. Currently, products (honey, royal jelly, propolis, beeswax or bee pollen) are gaining prominence due to the presence of bioactive compounds that are associated with health-beneficial properties [6,7].

Bee pollen is gaining attention as a functional food for human consumption due to its high content of compounds with health promoting effects such as essential amino acids, enzymes, fatty acids, antioxidants, vitamins, minerals, lipids, carbohydrates, and polyphenols [7,8]. It is used as an alternative and complementary therapy to cure prostatitis, stomach ulcers and infectious diseases. A wide range of therapeutic properties have been suggested, such as antimicrobial, antioxidant, hepatoprotective, chemopreventive and anti-carcinogenic, anti-atherosclerotic, anti-inflammatory, anti-allergic and immunomodulatory [7,9,10,11].

*Melipona fasciculata* Smith 1858 (Apidae, Meliponini), is a species of stingless bee, traditionally cultivated in the state of Maranhão, Brazil, by small and medium producers primarily for the production and marketing of honey [12]. However, other products of this species, such as pollen can be explored, being an important product with potential for chemical and biological exploitation.

The anti-inflammatory pollen and bee pollen activity has been described both in vitro and in vivo models. Lee et al. [13] investigated the effects of pollen extract on the modulation of pro-inflammatory mediators production in RAW 264.7 macrophages lipopolysaccharide-activated (LPS) and observed inhibition in the production of nitric oxide (NO), tumor necrosis factor-α (TNF- α), IL-1 and IL-6. Studies have shown that pollen’s anti-inflammatory activity is due to the presence of phenolic compounds such as flavonoids and phenolic acids and fatty acids and phytosterols. Kaempferol, which is a flavonoid that inhibits the activity of two enzymes: hyaluronidase, which is an enzyme that catalyzes the depolymerization of hyaluronic acid and elastase, which hydrolyses elastin, strengthens connective tissue and seals blood, resulting in decreased transudates, reactions. inflammatory and edema. It has also been demonstrated that the anti-edematous, anti-inflammatory and analgesic action of quercetin, which inhibits the activity of histidine decarboxylase, reduces the level of histamine in the organism, and may also inhibit the cascade of arachidonic acid metabolism, decreasing the level of pro-inflammatory prostaglandins, having an anti-inflammatory effect due to eliminating local pain and preventing platelet aggregation [14,15]. 

Our research group previously reported that extracts from *M. fasciculata* geopropolis have antioxidant activity [16,17], leishmanicidal [18], antimicrobial and immunomodulatory [19] anti-tumor [20] and anthelmintic [21], but there are no records regarding the anti-inflammatory and antinociceptive activity of pollen collected by *M. fasciculata*. Considering the bioactivity and chemical composition of the pollen of honey bees and stingless bees of the genus *Melipona*, especially *M. fasciculata*, we support the hypothesis that pollen collected from *M. fasciculata* contains bioactive substances that can be used to treat pain and inflammation. 

Thus, this study evaluated the anti-inflammatory and antinociceptive activity and the hydroethanolic pollen extract collected by *M. fasciculata* from different places, identified the compounds present in the material and correlated which compounds are associated with bioactivity through in silico, in vitro and in vivo assays.

## 2. Results

### 2.1. Total Phenolic Content, Total Flavonoids Content and Antioxidant Activity

The Total Phenolic Content, Total Flavonoids Content and Antioxidant Activity (DPPH•, FRAP and ABTS^•+^ methods) are present on Table 1. In this moment, was utilized four samples from Palmeirândia (EHPP1–4), three samples from Viana (EHPV1–3) and one sample from Chapadinha (EHPC) cities of pollen extract collected by M. fasciculata. The TPC ranging to 6.10% to 11.40%. The TFC ranging to 0.30 to 2.09%. The extract that show highest total phenolic content, total flavonoid content and minor DPPH• IC_50_ is the extract from EHPC, followed by the EHPP1 and EHPP3. Regarding the FRAP and ABTS^•+^ assays, the extracts with the highest potential were EHPP1, EHPC and EHPP3. 

### 2.2. COX–1 and 2 Inhibition Assay

**Figure 1:**
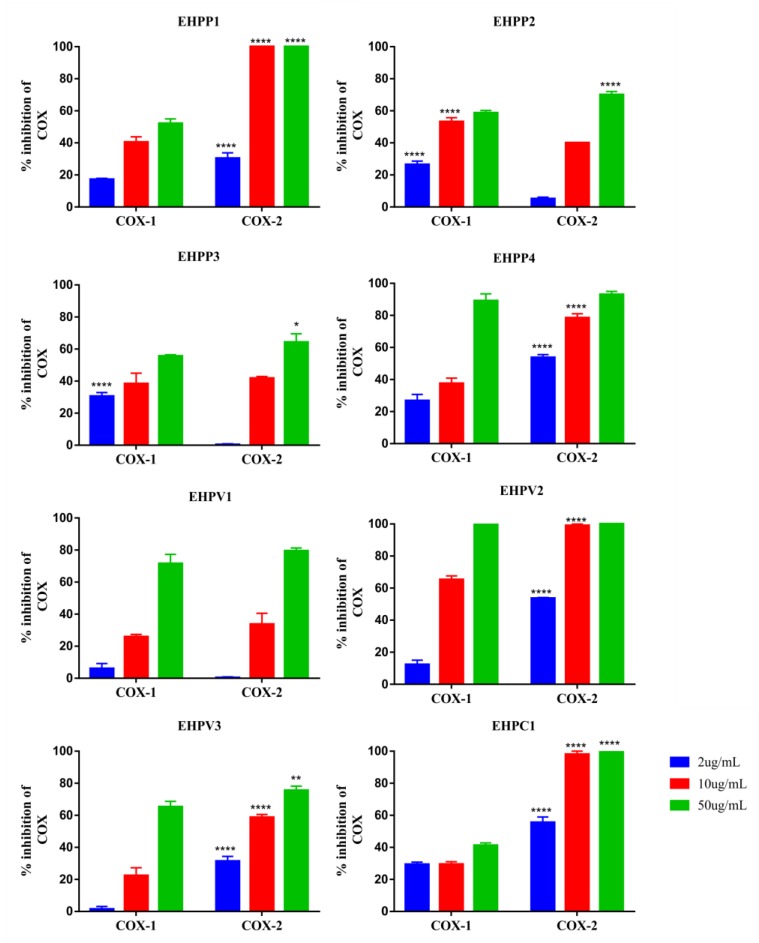
Percentage of in vitro inhibition of COX-1 and 2 produced by hydroethanolic pollen extract collected by *M. fasciculata* was obtained in Chapadinha (EHPC), Palmeirândia (EHPP1-4) and Viana (EHPV1-3) municipalities. Tested at three concentrations: 2 μg/mL, 10 μg/mL and 50 μg/mL. * Represents significant differences, * with *p* < 0.05; ** with *p* < 0.005; **** with *p* < 0.0001 comparing inhibition of cyclooxygenase 1 (COX-1) with 2 (COX-2). (Two-way ANOVA; Sidak).

All extracts were evaluated for inhibition potential of COX-1 and 2 enzymes. EHPC was the extract that showed the best activity, inhibiting 100% of COX-2 and only 27% of COX-1 at 10 µg/mL. EHPP1 extract also inhibited 100% of COX-2, but inhibited COX-1 by 38% also at 10 µg/mL, a value about 30% higher than that observed in EHPC. The other extracts tested strongly inhibited both enzymes (EHPV2 and EHPP4) or had a maximum inhibitory activity of less than 75% (EHPP2-3; EHV1-3) at a concentration of 50 µg/ mL (Figure 1).

Due it had the best TPC, TFC, DPPH• IC_50_, was the second best in FRAP and ABTS*^•+^* results, and had a higher affinity for COX–2 over COX–1, we chose EHPC for the posterior in vivo phase of the study and chemical characterization.

### 2.3. In Vivo Anti–Inflammatory Activity

#### 2.3.1. Carrageenan–Induced Paw Edema Test

**Figure 2:**
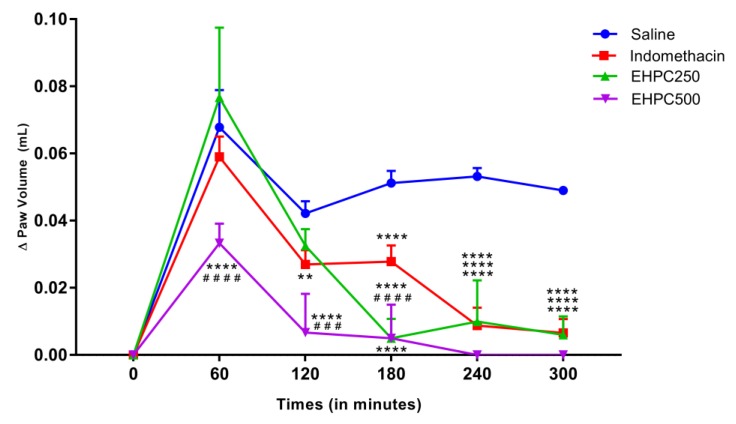
Carrageenan–Induced Paw Edema by subplantar administration of 1% carrageenan in orally treated mice with 0.9% NaCl, indomethacin 10 mg/kg, EHPC 250 and 500 mg/kg. ** *p* < 0.01; **** *p* < 0.0001 vs. CTRL; ### *p* < 0.001; #### *p* < 0.0001 vs. Indomethacin (ANOVA; Tukey).

Treatment with EHPC at 500 mg/kg significantly reduced mice paw edema in 52% compared with the saline group from the first hour of test (*p* < 0.0001). Thereafter, these treatments reduced edema by 89%, 94%, and 100% at 2, 3, and 4 h, respectively (*p* < 0.0001 at all times) compared to saline. Treatment with the 250 mg/kg dose of EHPC no showed statistical difference in reducing edema within 1 and 2 h, but was able to reduce edema by 94%, 91%, and 100% at 3, 4, and 5 h, respectively, being statistically different from the saline group (*p* < 0.0001). Treatment with EHPC 250 mg/kg showed a statistically significant reduction in edema reduction 3 h after induction compared with indomethacin (*p* < 0.0001) and provides statistically similar effects for this drug at 4 and 5 h. The treatment with 500 mg/kg of EHPC, was significant (*p* < 0.001) better than indomethacin in the first three hours of evaluation and statistically equal in the last two hours (Figure 2).

#### 2.3.2. Dextran–Induced Paw Edema Test

**Figure 3:**
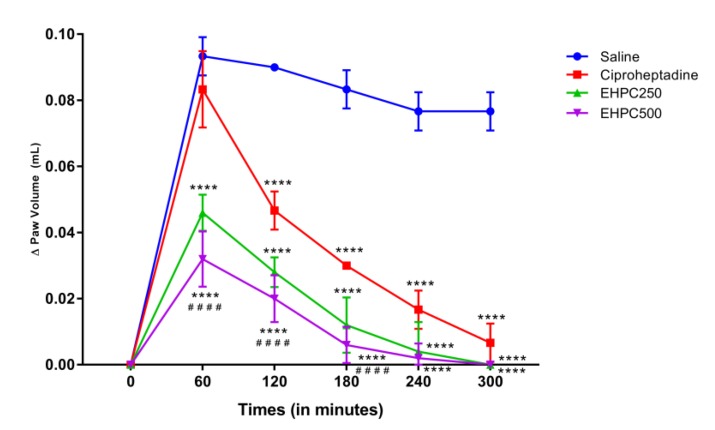
Dextran–Induced Paw Edema induced by subplantar administration of 1% dextran in mice treated orally with 0.9% NaCl, ciproheptadine 10 mg/kg, EHP 250 mg/kg and EHP500 mg/kg. **** *p* < 0.0001 vs. CTRL; #### *p* < 0.0001 vs. ciproheptadine (ANOVA; Tukey).

EHPC treatment at both doses was statistically different from the saline group at all-time points, as shown in Figure 3. The reduction in paw edema in the EHPC 250 mg/kg group from 1 to 5 h varied between 52% and 100% compared to the saline group; and in the EHPC 500 mg/kg treated group the reduction in paw edema ranged from 66% to 100% over this same time period compared to the saline group. Animals treated with both doses of EHPC also showed greater efficiency in reducing edema than the group treated with standard drug cyproheptadine. EHPC groups 250 and 500 mg/kg significantly better than the drug on 1 to 3 h after induction (Figure 3). In the first hour of evaluation, the 500 mg/kg EHPC was 84% more efficient in reducing edema than cyproheptadine (Figure 3). Thus, demonstrating that the EHPC interferes more powerfully than the drug, acting mainly in the first hours of induction of the inflammatory process.

### 2.4. In Vivo Anti–Nociceptive Activity

#### 2.4.1. Acetic Acid Writhing Test

**Figure 4:**
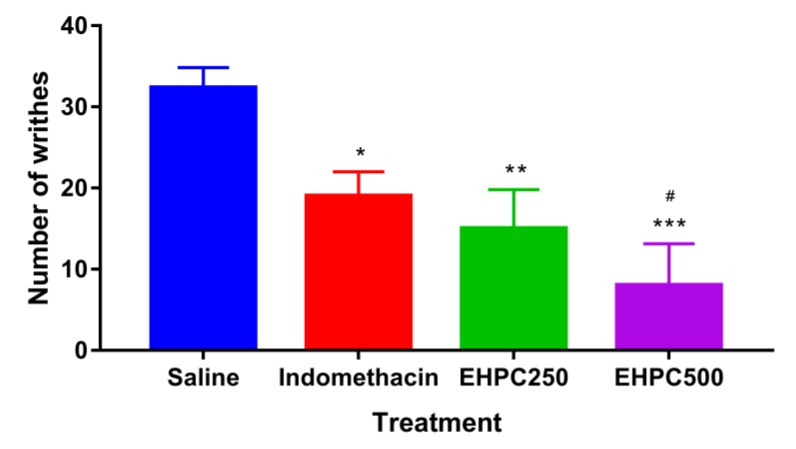
Writhing induced by the intraperitoneal administration of 0.8% acetic acid (10 mL/kg) in oral mice treated with 0.9% NaCl, indomethacin 10 mg/kg, EHPC 250 and 500 mg/kg. * *p* < 0.05; ** *p* < 0.01; *** *p* < 0.001 vs. NaCl; # *p* < 0.05 vs Indomethacin (ANOVA; Tukey).

Using orally administered EHPC extract at concentrations of 250 and 500 mg/kg, the number of abdominal contortions produced by intraperitoneal administration of 0.8% acetic acid solution in animals was significantly reduced by 54% (*p* < 0.01) and 76% (*p* < 0.001), respectively, compared with saline. There was no statistical difference between the decrease in the number of writhing between the indomethacin and EHPC250 groups, but we found that pollen extract at a concentration of 500 mg/mL was more efficient than indomethacin, causing 58% less abdominal writhing (*p* < 0.05). The results of writhing test are showed in Figure 4.

#### 2.4.2. Formalin Test

**Figure 5:**
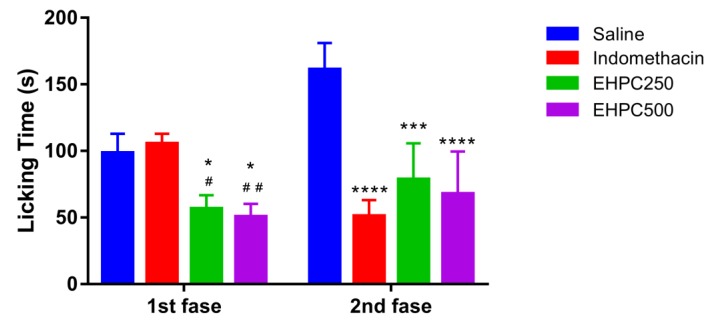
Formalin test induced by subplantar administration of 2.5% formalin in mice treated orally with 0.9% NaCl, indomethacin 10 mg/kg, EHPC 250 and 500 mg/kg. * *p* < 0.05; *** *p* < 0.01; **** *p* < 0.001 vs. NaCl; # *p* < 0.05; ## *p* < 0.005 vs Indomethacin (ANOVA; Tukey).

In the neurogenic phases (0–5 min) formalin-induced pain test, treatments using EHPC orally administered at concentrations of 250 and 500 mg/kg, decreases the time that the animals passed licking/biting the induced paw in 42% and 47%, respectively, generating statistically significant results when compared to the saline group (both *p* < 0.05). Compared with indomethacin, the extract also showed a significant reduction, where 250 mg/kg reduced 46% more than indomethacin (*p* < 0.05) and 500 mg/kg reduced 52% more than the standard drug (*p* < 0.005). In the inflammatory phases (15–30 min), the extract in 250 and 500 mg/kg doses reduces the inflammation levels in 52% (*p* < 0.01) and 59% (*p* < 0,0001), respectively, in comparison with saline. Indomethacin reduces the inflammation on 67%, statistically differing from saline. Also, the anti-inflammatory effect of pollen extracts was not statistically significant different from indomethacin (Figure 5).

### 2.5. LC-ESI-IT-MS/MS Analysis

**Figure 6:**
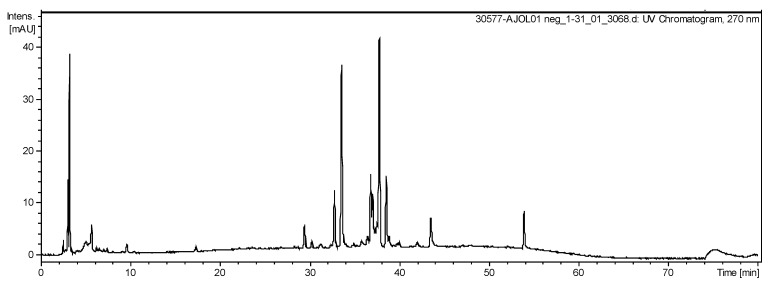
HPLC fingerprint (270 nm) of the hydroethanolic pollen extract collected by *M. fasciculata* from Chapadinha—MA.

**Figure 7:**
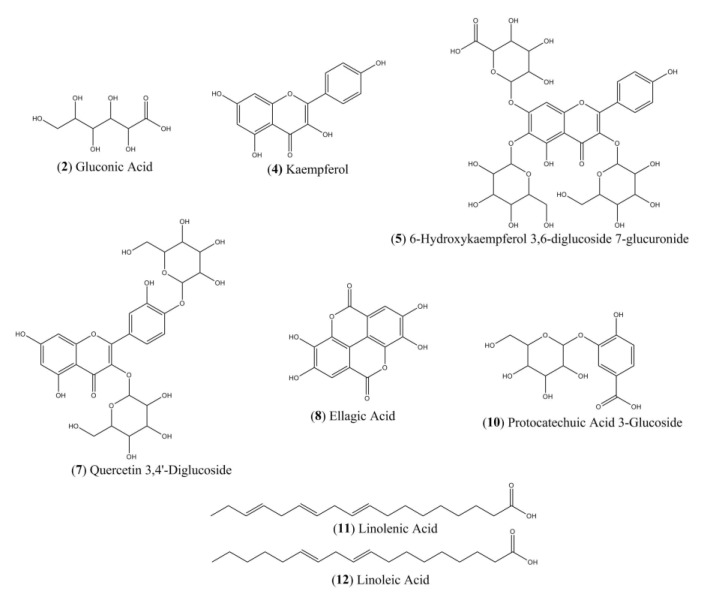
Chemical structures of the compounds identified by LC-ESI-IT-MS/MS in the hydroethanolic pollen extract collected by *M. fasciculata* from Chapadinha—MA.

The HPLC chromatogram showed a number of peaks corresponding EHPC to organic acids and phenolic compounds (Figure 6). Table 2 summarizes the molecular weight, molecular ion [M-H]^−^ and major product ions obtained by LC-MS/MS for 10 EHPC fragmentation peaks. The compounds (Figure 7) were identified by comparing their fragmentation profiles with the compounds described in the literature data.

### 2.6. Molecular Docking

**Figure 8:**
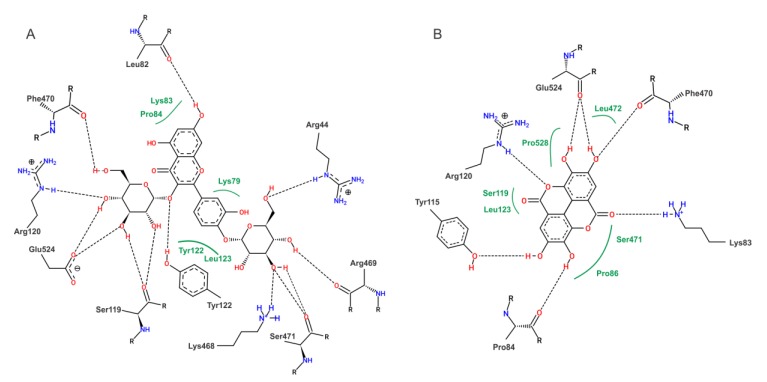
2D representation of the interactions of COX-2 residues with quercetin 3, 4’-diglucoside (**A**) and ellagic acid (**B**). Dashed lines—represent hydrogen bonds; green lines—van der Waals interactions.

To our molecular docking analysis, we used all compounds identified by HPLC-MS/MS on EHPC hydroethanolic extract. On general, the phenolic compounds showed highest affinity parameters on COX-2. The quercetin 3,4’-diglucoside and ellagic acid were the compounds that showed the best energy affinity parameters with COX-2, with binding free energy values of -8.13 and -7.60 kcal/mol and 1.11 and 2.68 µM inhibition constant, respectively (Table 3). In to the compounds present in the extract, the molecular docking of commercial NSAID Meloxicam was also performed. The results of meloxicam were close to quercetin 3,4’-diglucoside and ellagic acid parameters. The interactions performed by quercetin 3,4’-diglucoside and ellagic acid with the amino acid residues of the active site of COX-2 are shown in Figure 8. 

**Figure 9:**
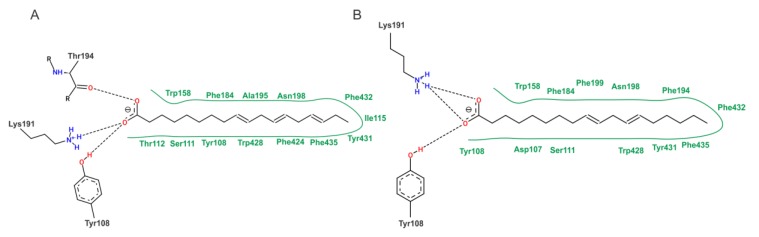
2D representation of the interactions of H1 histamine receptor residues with linolenic (**A**) and linoleic acids (**B**). Dashed line—represent hydrogen bonds; green lines—van der Waals interactions.

Regarding the type H1 histamine receptor, the compounds that presented the best electronic affinity parameters were linolenic and linoleic acids, being binding free energy values were −9.15 and −8.72 kcal/mol, respectively (Table 3). In addition, we perform the redocking of the Doxepin, ligand found in the original structure of H1 histamine receptor, using the same conditions as the other ligands to validate our protocol. The value of the free binding energy was −10.06 kcal/mol and the root mean square deviation (RMSD) between the predicted docking conformation and the observed X-ray crystal structure was 0.21 Å. Values below 2 Å indicate that the docking protocol is valid. The interactions performed by linolenic and linoleic acids with the amino acid residues of the active site of H1 histamine receptor are shown in Figure 9.

## 3. Discussion

Besides all extracts evaluated, the EHPC was the extract that show most efficient antioxidant activity. Our DPPH^•^ IC_50_ is lower compared to *Apis mellifera* bee pollen extract that show DPPH^•^ IC_50_ ranging 810 to 4690 µg/mL [22]. Also better to the DPPH^•^ IC_50_ found using the bee pollen extract from Portugal [23] and the bee pollen extract from Turkey [24]. However, samples of the *M. fasciculata* geopropolis extract showed much better DPPH^•^ IC_50_ than those found in the EHPC [16,17]. The presence of higher levels of phenolic and flavonoid compounds found in geopropolis may justify this difference. Our FRAP and ABTS^•+^ results of EHPC is according with previous studies using pollen extract [25,26]. Extracts with antioxidant properties have metabolites that have free radical scavenging properties and inhibit enzymes xanthine oxidase and lipoxygenase [27]. The interaction of these natural antioxidants with reactive oxygen species involved in the evolution of the inflammatory process has therefore encouraged several studies on the effects of these antioxidants on the formation of pro-inflammatory eicosanoids derived from COX arachidonic acid metabolism. Thus, according to our results, the EHPC has encouraging antioxidant activity, evidenced in the results of DPPH^•^ radical sequestration and iron reducing potential (FRAP) and ABTS^•+^ results 

COX is a key enzyme for inflammatory mediators’ biosynthesis, as it acts in the metabolization of arachidonic acid, leading to the production of prostaglandins, prostacyclin’s and thromboxane’s. In addition to pathological processes such as inflammation, various physiological processes are also involved. COX isoforms are classified as COX-1—with constitutive role, inducible COX-2 - linked to inflammatory processes, in addition to COX-3, which is a variant of COX-1 [28,29,30]. In the present study, all pollen extracts collected by *Melipona fasciculata*, when evaluated on in vitro inhibition assay, were capable of producing an inhibitory effect on COX-1 and COX-2, but the best results were expressed by the EHPC, which inhibited 100% to COX-2, and only 27% to COX-1 at a concentration of 10 µg/mL, thus being the extract with the highest affinity and most effective in inhibiting COX-2. Similar results were also reported in a study with *Cistus* spp. ethanolic bee pollen extract, which was also more selective for COX-2, however doses of 10 µg/mL reduced by 50% [31], and in our study, equivalent doses of EHPC reduced 100%, showing that our pollen extract was more efficient. These data indicate that the anti-inflammatory mechanism of action of the EHPC involves COX-2 inhibitory activity and consequent reduction of prostaglandin synthesis. Studies by Lee [32] also show that the anti-inflammatory effect of bee pollen is due to COX-2 inhibition through gene suppression.

Our investigations of the anti-inflammatory effect of EHPC showed that the 500 mg/kg dose significantly reduced carrageenan-induced paw edema from 1 h to 5 h, with reductions ranging from 52% to 100% of edema. The dose of 250 mg/kg also reduced the edema, however only differed statistically from the saline group from 3h, and by 5h had reached 100% reduction. These results prove the anti-inflammatory action of EHPC, since the carrageenan-induced paw edema model induces a powerful two-stage inflammation, the first occurs within 1h of carrageenan administration and cytoplasmic enzymes, histamine, are released and serotonin from mast cells. In the second phase (1–6 h) there is an increase in prostaglandin production by COX-2 activation and NO release and continuity between phases is provided by kinins [33,34]. The results of the present study also allow us to state that the EHPC interferes with both phases of inflammation. As is also the case with the bee pollen ethanolic extract from *Cistus* spp. (100 and 300 mg/kg) which significantly reduced carrageenan-induced edema in 1, 3, 4 and 5 h after induction [31]. Based in our results, a possible hypothesis from extract activity is that the EHPC that may act also as H1 histamine receptor antagonist and act on the COX-2 inhibition, thus impossibility the progression of the inflammatory process.

The anti-inflammatory effect of EHPC was also demonstrated in the dextran-induced paw edema model, in which the 250 and 500 mg/kg dose of EHPC reduced the edema from 1 to 5 h after evaluation. This model is characterized by increased vascular permeability due to mast cell degranulation with histamine and serotonin release [35]. That way, a possible explanation for the anti-edematogenic effect of EHPC could probably be due to a blocking effect on the synthesis, release or activation of vasoactive amines (histamine and 5-HT), causing possible vasoconstrictor activity. As proposed by Vasconcelos [36] by evaluating the effect of *Erythrina velutina* hydroethanolic extracts, which was also able to significantly inhibit dextran-induced paw edema at all times, however reaching a maximum of 51.3% inhibition of 400 mg/kg, while our extract (EHPC) has reduced by up to 100% at a dose of 250 mg/kg.

In this study, the antinociceptive activity of the EHPC was evaluated using the acetic acid-induced writhing test and the formalin test in mice. With the test of abdominal writhing induced by acetic acid, it was found that the extract was able to reduce the number of writhes by up to 76% at the dose of 500 mg/kg, being more effective than indomethacin. Acetic acid-induced abdominal writhing testing is a sensitive method for assessing peripheral action analgesics. Acetic acid induces the analgesic effect indirectly via endogenous mediators present in inflammatory processes such as bradykinin, serotonin, histamine, substance P and prostaglandins, which stimulate peripheral nociceptive neurons that respond to anti-inflammatory drugs [37,38]. Our hypothesis in the present study is that the antinociceptive action of the extract occurs through the peripheral reduction in prostaglandin synthesis, caused by the inhibition of cyclooxygenases as suggested by Abdulmalik et al. [39] when verifying the antinociceptive effect with the same pain model in the *Ficus iteophylla* leaves extract.

Studies conducted with propolis ethanolic extract also reported similar antinociceptive results, with a reduction in the number of writhing at doses of 100, 200 and 400 mg/kg by 21.73%, 38.96% and 40.68%, respectively [40]. However, EHPC results observed in the present study were considerably higher. 

The present study also indicated that the hydroethanolic pollen extract collected by *M. fasciculata* has analgesic properties not only on the peripheral but also on the central nervous system. Since in the formalin test the EHPC 250 and 500 mg/kg decreases the time animals spent licking/biting the induced paw by 42% and 47%, respectively, in the first phase, called the neurogenic phase, or early phase goes from zero to five minutes, which is the result of direct nociceptor stimulation and reflects central pain [41]. In the second phase, EHPC (250 and 500 mg/kg) also reduced inflammation levels by 52% and 59% compared with saline. This phase, which lasts from 15 to 30 min is called the inflammatory phase, and is characterized by local inflammation with release of inflammatory mediators and hyperalgesia [42].

Thus, the action of the extract in the first phase, reducing paw licking time is typical of an action demonstrated by centrally acting drugs (opioids) such as morphine, which also produces effects in the second phase [43]. However, the effect of the extract in the second phase may reflect not only the central action of the extract, but also a peripheral action, by inhibiting biosynthesis of mediators responsible for inflammation, such as inhibition of cyclooxygenase and consequently prostaglandins, such as It was also suggested by Moniruzzaman et al. [43] who reported that *Adenanthera pavonin* ethanolic leaf extract inhibited nociceptive responses in both phases of the formalin test. Choi [44] also found that the *Pinus* spp., pollen ethanolic extract (100 and 200 mg/kg, orraly) produced a significant inhibition of both phases of the formalin pain test in mice, and the author suggest that the different polyphenols found in pine pollen could be responsible for antinociceptive and anti-inflammatory activity’s. 

In the chemical analysis, we found phenolic, flavonoid, and fatty acids in the EHPC. Phenolic and flavonoid compounds are commonly reported with various biological activities, among which we highlight anti-inflammatory activity, which is usually associated with the ability of these compounds to modulate cellular components that participate in the mechanism of inflammation, such as pro-cytokines. such as TNF-α and IL-1, and inhibition of the activity of enzymes involved in the arachidonic acid pathway such as cyclooxygenase and lipoxygenase [45,46].

According our results from anti-inflammatory and antinociceptive activities from EHPC that suggests the H1 histamine receptor and COX-2 were a possible pathway of extract action, we also performed the molecular docking of the compounds identified in the EHPC against these targets.

Our results suggest that the fatty acids linolenic and linoleic acid are the compounds that have the best interaction parameters with the histamine H1 receptor. Linolenic acid was able to inhibit histamine release in RBL-2H3 cells [47]. Both fatty acids were also effective in preventing histamine release by rat peritoneal mast cells [48]. Also reported that the linoleic acid did not have an effect on lipid peroxidation and no show radical scavenging activity, indicating that this molecule no have antioxidant activity [49]. Regarding to COX-2, the quercetin 3,4’-diglucoside and ellagic acid was the compounds that have the best interaction parameters. Few papers report the biological activity from quercetin 3,4’-diglucoside, but this compound was shows potential antioxidant activity (including DPPH• and FRAP) [50,51] anti-urolithics, anti-urease pathogenesis, and anti-gout activity [51] anti-platelet aggregation [50], was inhibitor of α-amylase, α-glucosidase, acethyl and butyrylcholinesterase, anti-diabetic and could inhibit the proliferation of cancer cell lines [52]. The ellagic acid were described with a cornucopia of activities like antioxidant, anti-hepatotoxic, anti-steatosic, anti-cholestatic, anti-fibrogenic, anti-hepatocarcinogenic and antiviral properties [53]. The anti-inflammatory activity from ellagic acid were also reported, and the commonly attributed to reduction of NO, IL-1β, TNF-α, COX-2 and NF-κB [54]. These findings reinforce our hypothesis, built on the data from the present study, that these molecules have the potential to be the target of research for new drugs with anti-inflammatory and antinoniceptive activity.

## 4. Materials and Methods 

### 4.1. Obtaining Pollen and Preparing Extracts

**Figure 10:**
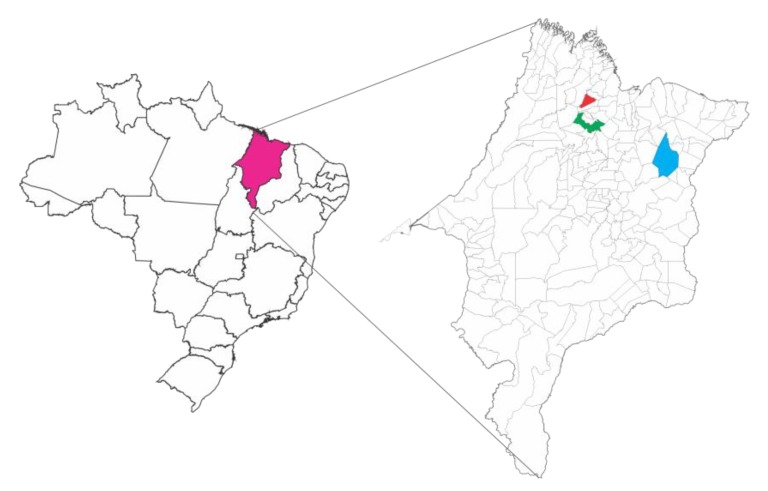
Localization of Maranhão State (in magenta) and the cities of Chapadinha (in blue), Palmeirândia (on red) and Viana (on green).

The samples of pollen collected by stinglees bee *M. fasciculata* was obtained in the municipality of Chapadinha (Cerrado, Brazilian savannah, Brazil), Palmeirândia and Viana (Baixada Maranhense, flooded fields area, Brazil) (Figure 10) in the state of Maranhão, Brazil, being taken directly from the beehives in the stingless beehives of these cities. After collection, pollen samples were identified, placed in a sterile container and kept refrigerated at 4 °C until use. The present research is registered on National System of Genetic Heritage Management and Associated Traditional Knowledge (SISGEN) under code AD841D2.

The pollen samples were individually extracted by maceration with 70% ethanol/water (70:30, *v*/*v*) with a solid to solvent ratio of 1 to 5 (*m*/*v*) for 72 h, with solvent renewal every 24 h. The resulting product from the three extractions was combined, filtered and concentrated in a rotary evaporator under vacuum at 40 °C, thus obtaining the pollen hydroethanolic extracts, which were coded for EHPC (Chapadinha sample), EHPP 1 to 4 (Palmeirândia samples) and EHPV 1 to 3 (Viana samples) and kept refrigerated until their use.

### 4.2. Determination of Total Phenolic Content (TPC) in Hydroethanolic Pollen Extracts

The total polyphenol contents in the extracts were determined using Folin–Ciocalteau reagent and 20% sodium carbonate (NaCO_3_). The reaction mixture was kept in the dark for 2h at room temperature and absorbance was measured at 760 nm using Lambda 35 UV–Vis spectrophotometer (Perkin Elmer, Inc., Waltham, MA, USA). The TPC was calculated from a gallic acid calibration curve (2,5–40.0 μg/mL) and expressed as gallic acid equivalent (%). Analyses were performed in triplicate and the mean value was calculated for each sample [16].

### 4.3. Determinations of Total Flavonoid Concentration (TFC) in Hydroethanolic Pollen Extracts

For total flavonoid concentration we used photocolorimetric method with 5% methanolic aluminum chloride solution (AlCl_3_). The mixture was kept in the dark for 30 min at room temperature and absorbance was measured at 425 nm in UV–Vis Lambda 35 spectrophotometer (Perkin Elmer, Inc., Waltham, MA, USA). The concentration was calculated from the calibration curve constructed with standard quercetin solution (Merck, Darmstadt, Germany) (1–30.00 μg/mL) and expressed as quercetin equivalent (%). The analyses were performed in triplicate [55].

### 4.4. Determination of Antioxidant Activity

#### 4.4.1. DPPH• Radical Scavenging Activity

The antioxidant activity of hydroethanolic pollen extracts was evaluated by using the DPPH• free radical scavenging assay as described by Brand–Willians et al., [56] with modifications from Dutra et al. [16]. The samples pollen extracts were diluted on methanol at different concentrations (30–480 µg/mL) and added to a methanol solution of DPPH• (40.0 μg/mL). After 30 min of reaction at room temperature in the dark, the absorbance of each solution was read at 517 nm in a Lambda 35 UV−Vis spectrophotometer (Perkin–Elmer, Inc., USA). Methanol was used as the control, and DPPH• solution was used as the blank. The percent inhibition was calculated using the formula

DPPH• scavenging activity (%)

100 − [(A_sample_ − A_blank_) × 100/A_control_] (1)

where A_sample_ = absorbance of the sample after 30 min of reaction, A_blank_ = absorbance of the blank, and A_control_ = absorbance of the control.

The percentage of scavenging activity was plotted against the sample concentration to obtain the IC50, defined as the concentration of sample necessary to cause 50% inhibition. All experiments were done in triplicate.

#### 4.4.2. Ferric Reducing Antioxidant Power Assay (FRAP)

This protocol was used to determine the antioxidant activity based on iron reduction using the FRAP assay. FRAP measures the ferric–reducing ability of a sample in acid medium (pH 3.6), forming an intense blue color as the ferric tripyridyltriazine (Fe^3+^−TPTZ) complex is reduced to the ferrous (Fe^2+^) form. The test was performed according Benzie et al. [57] with modifications from Dutra et al. [16]. The samples of pollen extracts were diluted on methanol at different concentrations (12,5–200 μg/mL). The absorbance of the reaction mixture was read at 593 nm in a Lambda 35 UV−vis spectrophotometer (Perkin–Elmer, Inc., USA) using FRAP solution as a blank. The results were expressed as millimoles of Fe^2+^ per gram of sample. All experiments were done in triplicate.

#### 4.4.3. ABTS^•+^ Assay

The ABTS solution was prepared in water and potassium persulfate and kept in the dark room for 16 h before testing for the complete oxidation of ABTS^•+^ and the generation of the highly stable chromophore cation radical 2,2′–azino–bis(3 ethylbenzothiazoline–6–sulfonic acid) (ABTS^•+^) [58] with [59] modifications. The ABTS^•+^ solution was diluted with 70% ethanol/water (70:30, *v*/*v*) until the absorbance at 734 nm reached 0.7 ± 0.02. Readings were performed by reacting 1000–20 μg/mL of pollen extracts with the ABTS^•+^ solution. All studies were performed at least in triplicate monitoring the decrease in absorbance for 6 min; results reported corresponded to the % of remaining chromophores compared to conditions in the absence of antioxidants. The IC_50_ values were determined to each sample, using the formula:

Scavenging ability (%) = (1 − A_sample_/A_blank_) × 100 (2)

### 4.5. COX Inhibition Assay

The assay was performed according to the manufacturer’s recommendations (COX Colorimetric Inhibitor Screening—Cayman Chemical^®^, Ann Arbor, Michigan, USA). 96-well microplates were initially identified, and inhibition tests were performed in triplicate for each concentration tested (2, 10 and 50 µg/mL) of pollen extract and for the reaction controls: BW—control without enzyme; A—with enzyme only, without inhibitor. The 3 “BW” wells received 160 µL Tris–HCl buffer, 10 µL HEME and 10 µL solvent–70% ethanol/water (70:30, *v*/*v*), used to dilute samples. The wells “A1–A3” received 150 µL Tris–HCl buffer, 10 µL HEME, 10 µL enzyme (COX–1 or COX–2) and 10 µL solvent 70% ethanol/water (70:30, *v*/*v*). Wells with pollen extract received 150 µL of Tris–HCl buffer, 10 µL of HEME, 10 µL of enzyme (COX–1 or COX–2) and 10 µL of solvent–diluted 70% ethanol/water (70:30, *v*/*v*) pollen extract at concentrations 2, 10 and 50 µg/mL. After mixing all of these reagents in each well, the plate was gently shaken for a few seconds, followed by a 5 min incubation period. Subsequently, 20 µL of the colorimetric substrate solution was added to each well of the plate and then 20 µL of arachidonic acid, substrate of the COX–catalyzed enzyme reaction, was added to each well. The plates were shaken again and incubated at 25 °C for a further two minutes and then read at 590 nm.

### 4.6. Animals

The present study used 80 adult male *Mus musculus* mice, *Swiss* strain, with weights ranging from 25 to 35 g, which were procured from the Central Vivarium (Biotério Central) of Federal University of Maranhão (UFMA), São Luis, Brazil. Animals were placed in polyethylene boxes (*n* = 5 per box) and provided with free access to food and water in an environment with controlled temperature and 12/12 h light/dark cycle at 22 °C. This study was carried out in accordance with the recommendations of IASP Guidelines for the Use of Animals in Research and according with National Council for Animal Experimentation Control–CONCEA. The experimental protocols were approved in 16 January 2017 by the UFMA Ethics in Animal Use Committee (CEUA), ruling no. 64/2016, protocol no. 23115.016655/2016–83.

### 4.7. Anti–Inflammatory Activity

#### 4.7.1. Carrageenan–Induced Paw Edema Test

Mice were randomized to groups (*n* = 5) treated oral with vehicle (saline) (10 mL/kg), pollen extract (250 and 500 mg/kg) or indomethacin (10 mg/kg). After 60 min of treatment, paw edema was induced by subplantar administration of 50 µL of 1% carrageenan into the right paw of the animal. The paw volume of the animal was measured by the digital plethysmometer (Ugo Basile Model, Verese, Italy) during 0, 1, 2, 3, 4 and 5 h after induction [60,61]. The edema value was also obtained by the difference between the right paw volume in the respective hour comparing with the basal volume, being expressed as the variation of the paw volume (mL) over time.

#### 4.7.2. Dextran–Induced Paw Edema Test

The test was used to evaluate pharmacological activity from subplantar administration of 1% dextran. Mice were randomized to groups (*n* = 5) orally treated with vehicle (saline) (10 mL/kg), pollen extract (250 and 500 mg/kg) or Ciproheptadine 10 mg/kg. After 60 min of treatment, paw edema was induced by subplantar administration of 50 µL of 1% dextran into the right paw of the animal. The paw volume of the animal was measured by the digital plethysmometer (Ugo Basile Model, Verese, Italy) during 0, 1, 2, 3, 4 and 5 h after induction [35]. The edema value was also obtained by the difference between the right paw volume in the respective hour comparing with the basal volume, being expressed as the variation of the paw volume (mL) over time.

### 4.8. Anti–Nociceptive Activity

#### 4.8.1. Acetic Acid Writhing Test

The mice (*n* = 5) were treated orally with pollen extract (250 and 500 mg/kg), indomethacin (10 mg/kg) or vehicle (saline) (10 mL/kg) one hour before intraperitoneal (ip) administration of the acetic acid solution at 0.8% (10 mL/kg). The number of writhes was counted for each animal over a period of 20 min after administration of the acetic acid solution. Results were expressed as the average of the cumulative number of writhes [61,62].

#### 4.8.2. Formalin Test

The mice (*n* = 5) were treated orally with pollen extract (250 and 500 mg/kg), indomethacin (10 mg/kg) or vehicle (saline) (10 mL/kg) one hour before the subplantar injection of 20 μL of 2.5% formalin in the right paw. The nociceptive response, characterized by paw licking or biting, was observed during the first 5 min to assess neurogenic mechanisms and then from minutes 15 to 30 to assess inflammatory mechanisms [61,63].

### 4.9. HPLC– ESI–MS/MS Analysis

The pollen extract was analyzed by HPLC (LC–20AD Shimadzu, Kyoto, JP) and a Phenomenex Luna C–18 (250 × 4.6 mm-5 µm) column at 25 °C was used. The mobile phases consisted of ultrapure water containing 0.1% formic acid (A) and methanol (B). The following linear gradient was applied: 0 min, 5% B; 1−60 min, 5−100% B; 60−70 min, 100% B at flow of 1 mL/min. The LC was coupled to a mass spectrometer (Amazon Speed ETD, Bruker, Massachusetts, USA) equipped with electrospray ionization (ESI) and an ion–trap (IT) type analyzer in negative mode, under the following conditions: 4.5 kV capillary voltage, capillary temperature 325 °C, entrainment gas (N_2_) flow 12 L/min, nitrogen nebulizer pressure at 27 psi. The acquisition range was m/z 100–1000, with two or more events.

### 4.10. Computational Study

#### 4.10.1. Predictive Models and Theoretical Calculations

The compounds identified in the pollen extract from Chapadinha had their geometric, electronic and vibrational properties optimized using the Gaussian program 09 [64]. The GaussView 5.0.8 [65] was used to obtain 3D structural models. Geometric optimization calculations were performed according to the Functional Density Theory (DFT) method, combining the functional hybrid B3LYP and the set of bases 6–31 ++ G (d, p).

#### 4.10.2. Molecular Docking

All docking procedures utilized Autodock 4.2 package [66,67]. The structure of cyclooxygenase 2 (COX-2) (PDB ID 1DDX), H1-type histamine receptor (PDB ID 3RZE) and ligands were prepared for docking simulations with AutoDock Tools, version 1.5.6 [68]. Macromolecules had too many ligands and artifacts removed from their original files preserving only macromolecules. Docking methodology described in literature were used [69] with modifications [61,70]. Gasteiger partial charges were calculated after addition of all hydrogens. Non–polar hydrogens from COX–2, H1 histamine receptor and pollen extract compounds were subsequently merged. The dimensions of the cubic box in the X–, Y– and Z–axes were 70 Å × 70 Å × 700 Å, respectively, with a spacing of 0.375 Å between grid points. The grid box was centered on the oxygen atom of Arg120 residue from COX-2 structure and in the oxygen atom of Trp428 residue H1 receptor structure and Lamarckian genetic algorithm (LGA) was chosen to search for the best conformations, with 100 runs for each compound. Initial coordinates of COX–2 or H1 receptor and pollen extracts secondary metabolites interactions were chosen using the criterion of lowest docking conformation of cluster with lowest energy combined with visual inspection.

### 4.11. Statistical Analysis

Statistical analyzes between experimental groups were performed by analysis of variance (ANOVA) followed by Tukey test. The results that presented probability of occurrence of null hypothesis lower than 5% (*p* < 0.05) were considered statistically significant. Statistical analysis was performed using Graphpad Prima^®^ 7 software.

## 5. Conclusions

The hydroethanolic pollen extract collected by *M. fasciculata* shows great content of polyphenols, flavonoids, and antioxidant activity. The selected extract, also shows high inhibitory activity against COX-2, being this isoform inhibited 68% more that COX-1. The selected extract as well shows highly anti-inflammatory and antinociceptive activity. The in-silico results, in concordance to in vivo results, suggests that this activity can be due to action of the extracts compounds on histamine release inhibition and prostaglandins synthesis inhibition. Thus, we demonstrated for the first time these activities from the pollen collected by *M. fasciculata* with results higher compared to products to others bees, including *Apis mellifera*, and this evidence the potential use of stingless bee products in the development for new therapeutic agents.

## Figures and Tables

**Table 1:** Values of polyphenols and total flavonoids contents, activity antioxidant (DPPH•, FRAP and ABTS^•+^ methods) from pollen extracts collected by Melipona fasciculata Smith.

Extrato	CPT (%) ^a,b^	CFT (%) ^a,c^	DPPH• IC_50_ (μg/mL)	FRAP (mmol Fe^2+^/g)	ABTS^•+^ IC_50_ (μg/mL)
EHPP1	11.06 ± 0.08	1.47 ± 0.06	205.17 ± 0.08 ^c^	0.99 ± 0.05 ^b^	34.30 ± 0.22 ^b^
EHPP2	8.36 ± 0.82	0.94 ± 0.01	373.56 ± 1.32 ^d^	0.83 ± 0.08 ^b^	-
EHPP3	10.22 ± 0.54	1.16 ± 0.03	178.91 ± 1.09 ^e^	0.84 ± 0.09 ^b^	103.93 ± 0.14 ^d^
EHPP4	8.87 ± 0.22	0.65 ± 0.01	269.73 ± 0.05 ^f^	0.62 ± 0.05 ^c^	-
EHPV1	6.10 ± 0.31	0.40 ± 0.00	557.53 ± 0.61 ^g^	0.15 ± 0.08 ^e^	235.19 ± 0.19 ^f^
EHPV2	9.01 ± 1.05	0.35 ± 001	597.93 ± 0.96 ^h^	0.34 ± 0.03 ^d^	-
EHPV3	9.01 ± 0.02	0.30 ± 002	560.82 ± 0.20 ^i^	0.30 ± 0.03 ^d^	202.60 ± 0.15 ^e^
EHPC	11.4 ± 0.31	2.09 ± 0.02	117 ± 0.03 ^b^	0.84 ± 0.03 ^b^	70.77 ± 0.15 ^c^
Trolox	-	-	3.05 ± 0.61 ^a^	8.74 ± 0.13 ^a^	3.42 ± 0.41 ^a^

Values represent the mean of triplicate measurements ± standard deviation. Different letters in the same column indicate a significant difference (Tukey, *p* < 0.05). EHPP1–4, hydroethanolic pollen extract from *M. fasciculata* Smith, Palmeirândia-MA; EHPV1-3, hydroethanolic pollen extract from *M. fasciculata*, Viana-MA; EHPC, hydroethanolic pollen extract from *M. fasciculata*, Chapadinha-MA; (a) Results expressed as means ± standard deviation of quantitative evaluation tests for total polyphenols and total flavonoids in *M. fasciculata* pollen extracts (*n* = 3), (b) expressed as gallic acid equivalent; (c) expressed as quercetin equivalent. DPPH•, 2,2-diphenyl-1-picrylhydrazyl radical; FRAP, ferric reducing antioxidant power; ABTS^•+^, 2,2′-azinobis-3-ethylbenzotiazoline-6-sulfonic acid. (-) unrealized.

**Table 2:** Identification of compounds by LC-ESI-IT-MS/MS, in negative mode, of the hydroethanolic pollen extract collected by *M. fasciculata* from Chapadinha - MA.

Nº	Time Retention (min)	[M-H]^−^	MS^n^ Ion m/z (−)	Tentative Identification
1	2.9	539	195	gluconic acid derivate
2	3.0	195	177; 129	gluconic acid
3	29.4	571	285	kaempeferol derivative
4	29.5	285	255	kaempferol
5	30,1	801	539; 285	6-hydroxykaempferol 3,6-diglucoside 7-glucuronide
6	33.6	603	301	ellagic acid dimer
7	33.6	625	301	quercetin 3,4’-diglucoside
8	33.6	301	-	ellagic acid
9	36.8	1345	672; 522; 372	NI
10	36.9	315	299; 153	protocatechuic acid 3-glucoside
11	39.2	277	233; 179	linolenic acid
12	42.3	279	261	linoleic acid

NI—not identified.

**Table 3:** Free-binding energies (ΔGbind in kcal/mol) and inhibition constant (Ki, in µM) obtained by molecular docking of the compounds identified in the EHPC hydroethanolic extract with the COX-2 structure and H1 histamine receptor.

COX-2			Histamine H1 Receptor		
Ligand	ΔGbind (kcal/mol)	Ki (μM)	Ligand	ΔGbind (kcal/mol)	Ki (μM)
Quercetin 3,4’-diglucoside	−8.13	1.11	Linolenic acid	−9.15	0.18
Ellagic acid	−7.60	2.68	Linoleic acid	−8.72	0.40
Kaempferol	−7.44	3.54	Kaempferol	−8.32	0.80
6-Hydroxykaempferol 3,6-diglucoside 7-glucuronide	−7.07	6.57	Protocatechuic acid 3-glucoside	−7.49	3.25
Protocatechuic acid 3-glucoside	−6.91	8.59	Ellagic acid	−6.51	23.74
Linolenic acid	−6.62	13.99	Quercetin 3,4’-diglucoside	−2.31	201
Linoleic acid	−5.96	42.49	6-Hydroxykaempferol 3,6-diglucoside 7-glucuronide	−1.18	1370
Gluconic acid	−4.51	491.05	Gluconic acid	0.77	2719
Meloxicam	−8.63	0.49	Doxepin	−10.36	0.02

## Abbreviations

LPS	lipopolysaccharide
NO	Nitric oxide
TNF-α	tumor necrosis fator α
IL	Interleukin
DPPH•	2,2-difenil-1-picrilhidrazil
FRAP	Ferric reducing antioxidant power
ABTS	2,2′-azinobis-3-ethylbenzotiazoline-6-sulfonic acid
EHPP	Hydroethanolic pollen extract from Palmeirândia–MA, Brazil
EHPV	Hydroethanolic pollen extract from Viana–MA, Brazil
EHPC	Hydroethanolic pollen extract from Chapadinha–MA, Brazil
IC_50_	Concentration of sample necessary to cause 50% inhibition
COX	Cyclooxygenase
TPC	Total Phenolic Content
TFC	Total Flavonoid Concentration
NI	Not identified
HPLC	High performance liquid chromatography
LC-ESI-IT-MS/MS	liquid chromatography coupled to mass spectrometer with electrospray ionization and ion trap analyzer
RMSD	root mean square deviation
LGA	Lamarckian genetic algorithm
DFT	Density Functional Theory
UFMA	Federal University of Maranhão
CONCEA	National Council for Animal Experimentation Control
CEUA	Ethics in Animal Use Committee
AlCl_3_	Aluminum chloride
NaCO_3_	Sodium carbonate
5-HT	5-hydroxytryptamine
